# One-step sorting of single-walled carbon nanotubes using aqueous two-phase extraction in the presence of basic salts

**DOI:** 10.1038/s41598-020-66264-7

**Published:** 2020-06-08

**Authors:** Blazej Podlesny, Tomohiro Shiraki, Dawid Janas

**Affiliations:** 1 Department of Organic Chemistry, Bioorganic Chemistry and Biotechnology, Silesian University of Technology, B. Krzywoustego 4, 44-100 Gliwice, Poland; 2 Department of Applied Chemistry, Graduate School of Engineering, Kyushu University, 744 Motooka, Nishi-ku 819-0395 Fukuoka, Japan

**Keywords:** Nanoscale materials, Carbon nanotubes and fullerenes

## Abstract

We demonstrate a simple one-step approach to separate (6,5) CNTs from raw material by using the aqueous two-phase extraction method. To reach this goal, stable and inexpensive K_2_CO_3_, Na_2_CO_3_, Li_2_CO_3,_ and K_3_PO_4_ basic salts are used as modulators of the differentiation process. Under the appropriate parameters, near monochiral fractions become available for straightforward harvesting. In parallel, we show that the isolation process is strongly affected not only by pH but by the inherent nature of the introduced chemical species as well. The results of our study also reveal that the commonly used ingredients of the biphasic system make a strong contribution to the course of the separation by having far from neutral pH values themselves.

## Introduction

Although it has been almost 30 years since S. Ijima ignited the interest of the academic community in carbon nanotubes (CNTs)^1,2^, this materials is still at the focal point of numerous scientists from every corner of the globe. Every day, promising electrical^3,4^, mechanical^5^ and optical properties^6^ of these nanostructures encourage many research groups to work towards bringing nanocarbon to the masses. A lot is at stake because the unique characteristics of these materials make them a suitable antidote to a wide range of technological limitations of the civilization. Unfortunately, the time span of almost three decades spent on this mission so far indicates that the materialization of this dream is not trivial. The gravity of this challenge can be appreciated already by taking a closer look at the name of this material, which reveals its complexity. At present, synthesized CNTs are not simply a plurality of the exact copies of hollow carbon tubules of a particular structure, but the CNT powders, film and fibers are composed of mixtures of tens of CNT types of radically different properties from one another^7,8^. That is because the way how CNTs are conceptually rolled up from a graphene sheet (quantified by the so-called chiral angle) has a predominant effect on the properties of the resulting material. As shown by previous research, electrical^5^, mechanical^9^, thermal^10^ and optical^6,11^ properties of these nanoarchitectures are all highly dependent on this parameter. As a consequence, work with unprocessed raw material is characterized by somewhat averaged properties of individual CNT types, which very much restricts the depth of R&D conducted in this field. It has recently become clear that the post-synthetic sorting of CNTs is necessary to understand the nature of the material^12^ to eventually lay the foundation for its implementation.

To tackle this problem, over the past years, many separation methods have been established for the sorting of single-walled CNT (SWCNT) mixtures. The mainstream techniques are based on the concepts of selective solubilization with polymers^13–15^, chromatography^16,17^, electrophoresis^18^, density gradient ultracentrifugation(DGU)^19^ and biphasic extraction^20–25^. The latter technique commonly referred to as the aqueous two-phase extraction method (ATPE) has unveiled a particular promise on this front. It is rapid, simple to conduct, does not require sophisticated apparatus, and, most importantly, offers very high resolution. In the ATPE system, the components of the CNT mixtures are distributed between two immiscible phases. Stepwise physical separation of these phases and subsequent combination with fresh complementary top/bottom solutions of particular composition enables fractionation of the material. At present, such routine gives rise to the isolation of SWCNTs of particular electronic character^26,27^, chirality^23,28–30^ and even handedness^28^ both in the small-^21,24,29^ and since recently also in the large-diameter regime^28^.

For processing SWCNT mixtures, polymer-polymer systems are used most frequently, wherein the phases are formed by polyethylene glycol (PEG) and dextran (DEX)/polyacrylamide (PAM)^22,23^. Additional components are introduced such as sodium cholate (SC), sodium deoxycholate (DOC) or sodium dodecyl sulfate (SDS) to change the way how SWCNTs partition between the phases, which is the driving force for the separation. Commonly, the ATPE of SWCNTs is conducted in the multistep fashion to isolate the broadest possible spectrum of species one by one. We^30^ and other groups^22,29^ have recently started exploring the possibility of carrying out the separation in a reduced number of steps. For this purpose, we study the parameter space of the system to find the conditions able to yield particular SWCNT species ideally in a single step. It becomes more and more obvious that a wider nanocarbon community requires simple and robust protocols to obtain chirality-defined SWCNT fractions for various fields of exploitation without getting into the nuisances of separation. It has been observed that to make the sorting easy one commonly needs to introduce a chemical modulator into the biphasic system, which will enhance the otherwise minute differences between various CNT species. Then, under carefully selected conditions appropriate partitioning opens the route towards straightforward isolation of targeted CNT types. Up to date experience shows that chemicals for this task can either be redox^25^ or pH changing compounds^28^. We have recently shown that the molecule that plays a particularly helpful role for this purpose is ammonia, the introduction of which enabled us to separate SWCNTs dispersions of (6,5) light emission characteristics^30^ in one step. Despite the merits of this approach, ammonia, which is commonly employed as ammonia water can change concentration over time (particularly, if a significant portion of the container has been utilized). Since the ATPE system is very sensitive to the concentration of individual components, we noticed that variation in NH_3_·H_2_O concentration can affect the separation result.

In this contribution, we present how to avoid this problem by employing a palette of inorganic salts of basic character. The addition of potassium, sodium or lithium carbonates or phosphates conveniently increases the pH of the ATPE system to reach the desired partitioning in a similar way to the action of ammonia water. The modulating chemical compounds used in this contribution are very convenient since they are more stable in storage, not hazardous, inexpensive, but, most importantly, easy to employ even by non-specialists as compared to strong acids/bases, which need extra precautions. As a result, SWCNT fractions of (6,5) light emission characteristics emerge in a single step. For some experiments, enantiomeric excess of right- or left-handed isomers seem to take place. In parallel, we also demonstrate herein that common ingredients of the ATPE system have pH values, which are far from neutral, which should be taken into consideration while designing and executing SWCNT separation by the ATPE method.

## Experimental

## Chemical compounds

Dextran (DEX, M_R_ – relative molecular mass of ca. 70,000 Da, PanRecAppliChem, Germany), poly(ethylene glycol) (PEG, M_n_ – number average molar mass of ca. 6,000 Da, Alfa Aesar Germany), sodium cholate (SC, PanRecAppliChem, Germany) and sodium dodecyl sulfate (SDS, Sigma-Aldrich, USA) were all obtained from the shown vendors and had pure p.a. class.

As basic salt chemical modulators we examined: potassium carbonate (K_2_CO_3_, Avantor, Poland), sodium carbonate (Na_2_CO_3_, Avantor, Poland), lithium carbonate (Li_2_CO_3_, Avantor, Poland) and potassium phosphate (K_3_PO_4_, Avantor, Poland). The concentration of aqueous solutions prepared using these chemical compounds was 10 wt% for all basic salts except for lithium carbonate. Due to its reduced solubility in water, 1 wt% solution was made.

For this work, we engaged small-diameter unsorted SWCNTs HiPco Purified SWCNT material (batch HP30-006) purchased from NanoIntegris (Boisbriand, Canada).

In all cases when we used water, it was double-distilled in house using the Elix Millipore system. The electrical conductivity of the obtained water was cross-checked with a standard to ensure its suitability for this study.

### Preparation of SWCNT dispersions

SWCNT powder (40 mg) was introduced to a pre-prepared aqueous sodium cholate solution (2 wt%; 40 mL) kept in a 50 mL vial. This mixture was homogenized by ultrasound tip sonicator (Hielscher UP200St) for 2 h with a constant power of 50 W. It was kept an ice-bath during the sonication to ensure proper dispersion of the material. After homogenization, dispersion was immediately centrifuged (Eppendorf Centrifuge 5804 R) at constant temperature (18 °C) at the relative centrifugal force of 15,314 × g for 2 h. The upper 80% of supernatant was collected and used to further experiments.

### ATPE protocol

Aqueous solutions of PEG, DEX, SC, SDS (concentrations of 50 wt%, 20 wt%, 10 wt%, and 10 wt%, respectively) and alkaline modulator (concentrations of 10 wt% or 1 wt%) were transferred in the specified order to a centrifuge tube (5 mL) and gently homogenized by vortex mixer (VELP Scientifica) for 10 seconds. Next, CNT dispersion was added and the sample was homogenized again by vortex mixer. Obtained suspensions were centrifuged at a constant temperature (18 °C) at the relative centrifugal force of 2,025 × g for 3 minutes. After the centrifugation solution split into two phases, they were immediately collected by pipetting. In each experiment, the total volume of the ATPE system was 4.590 mL. Exact parameters of the separation routines are provided in the Supplementary Information file.

### Optical characterization

UV-VIS-NIR spectra were taken using the V-670 JASCO spectrophotometer. Excitation-emission photoluminescence (PL) maps were acquired using Horiba Jobin Yvon spectrofluorometer (Fluorolog-3 with FluorEssence) in the following wavelengths ranges: excitation (500–800 nm) and emission (900–1400 nm).

### pH measurements

For pH measurement, we used a digital pH-meter (VWR, PH20). Because all samples were prepared in air, measurement of pH was carried in the ambient once the samples reached equilibrium with atmospheric carbon dioxide. Standard error was assumed to be the resolution of the device declared by the manufacturer of ±0.1 pH unit. In practice, the deviation between measurement values was lower, but we decided to overestimate the uncertainty degree.

## Results and discussion

### Characterization of the starting material

**Figure 1:**
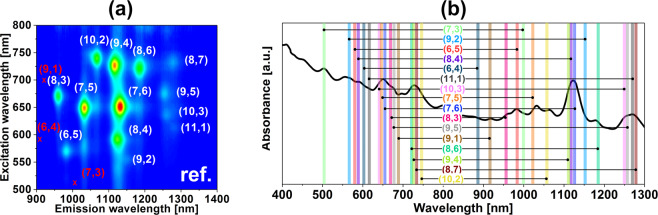
Characterization of the starting material (HiPco, unsorted SWCNTs). (**a**) 2D PL map (expected, but not detected CNTs marked in red), (**b**) absorbance spectrum with indicated optical transitions of the species detected in the study.

To get a detailed overview of the mechanics of ATPE separation, we selected unsorted SWCNTs produced by the HiPco process, which is known to have an assortment of CNT chiralities. In this particular sample, we detected the presence of 13 semiconducting species as visualized using 2D photoluminescence (PL) mapping (Fig. 1a). The diameter of constituting CNTs of this specific batch spanned from 0.629 nm (6,4) (*vide infra*) to 1.032 nm (8,7), which was to be expected from the synthesis technique routinely used to produce primarily small-diameter CNTs.

CNT chiralities identified by 2D PL mapping had their corresponding optical features in the recorded absorbance spectrum presented in Fig. 1b. We also noted the presence of three CNTs chiralities ((9,1), (7,3) and (6,4)) not observed by PL mapping bringing the total number of CNT semiconducting types up to 16. Judging on the absorbance spectrum we concluded that there were some CNTs of metallic character, which could not be seen by PL.

### Biphasic separation without chemical modulators

**Figure 2:**
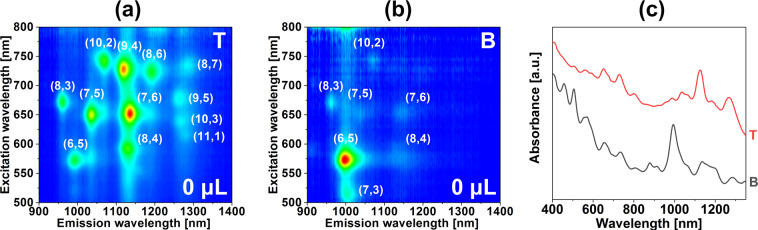
Characterization of the sorted material without the introduction of a chemical modulator. 2D PL maps of the (**a**) top, and (**b**) bottom phases. (**c**) Corresponding absorbance spectra. Conditions of the experiment can be found in Table S1.

We started the study by conducting a differentiation of SWCNT in the absence of basic salts by using the previously determined starting parameters^30^. As can be seen, these conditions already gave somewhat preliminary separation of CNTs (Fig. 2a,b). The dominant semiconducting CNT type in the bottom phase was (6,5) along with a significant content of metallic CNTs. Minor amounts of (10,2), (8,4) (8,3), (7,6), (7,5), and (7,3) were detected as well. All semiconducting CNTs had diameters below 0.9 nm. In this diameter regime, we also expected the presence of (9,2), (9,1) and (6,4) CNTs, the faint light signatures of which were discerned in the contour plots. They were more pronounced in the absorbance spectrum of the bottom phase wherein they manifested their characteristic S_11_ and S_22_ signatures (Fig. 2c).

The top phase, on the other hand, contained all the starting chiralities, and their ratios to some extent mimicked the composition of the parent material. The dominant chirality in this fraction was also (7,6). In this experiment, we used SC-dispersed CNTs and subjected them to SC/SDS competition. Similarly to the results shown above, Subbaiyan and colleagues reported that DOC-dispersed CNTs, when processed by the DEX-PEG ATPE system with SC/SDS/NaCl, exhibited a diameter cut^24^, which they used to isolate (6,5) CNTs in 2 steps. NaCl helped to shift large-diameter CNTs into the top phase due to its kosmotropic character^7^.

**Scheme 1:**
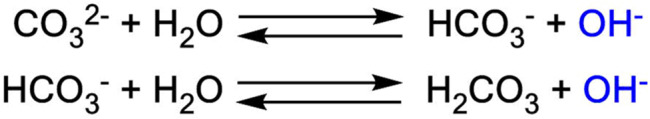
Two-step hydrolysis of a carbonate ion.

Recently, we observed that higher resolution may be obtained upon the addition of ammonia^30^. However, we noticed that over time the change in ammonia water concentration might hurt the result of ATPE separation of CNTs. This is supported by the results of previous studies which showed that even a 0.003% difference in surfactant concentration can affect the partitioning course^24^. To alleviate the problem of instability of NH_3_·H_2_O concentration in light of such remarkable sensitivity of the ATPE system, we decided to engage the following basic salts to tune the biphasic conditions: K_2_CO_3_, Na_2_CO_3_, Li_2_CO_3_, K_3_PO_4_. Acid dissociation constants of carbonate and phosphate are pKa_1_ = 6.4, pKa_2_ = 10.2 as well as pKa_1_ = 2.1, pKa_2_ = 7.2, pKa_3_ = 12.7, respectively, so they can readily increase the alkalinity of the medium due to the following phenomenon shown below for carbonate. Scheme 1.

**Figure 3:**
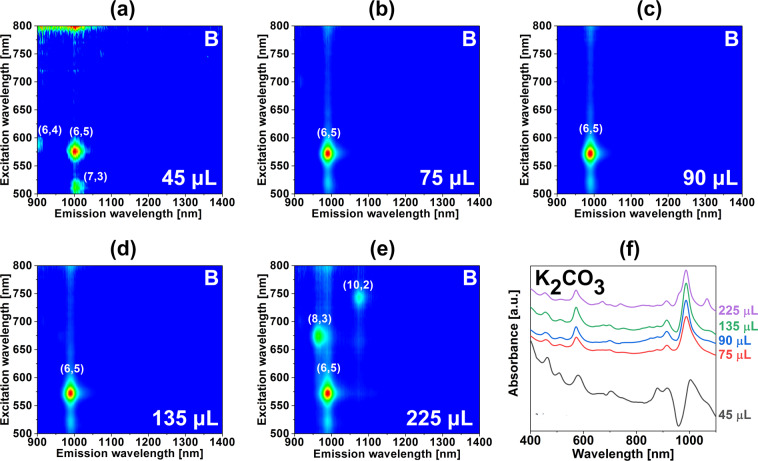
Characterization of the sorted material with the introduction of a chemical modulator (K_2_CO_3_) into the ATPE system. 2D PL maps of the bottom phases upon addition of (**a**) 45 μL, (b) 75 μL, (**c**) 90 μL, (**d**) 135 μL and (**e**) 225 μL of K_2_CO_3_ (10 wt%) per 4.59 mL total volume. (**f**) Corresponding absorbance spectra. Conditions of the experiments can be found in Table S2.

We began with the addition of a selection of volumes of K_2_CO_3_ (45 µL, 75 µL, 90 µL, 135 µL, and 225 µL). 2D PL analysis showed gradual changes to the way the CNTs distributed between two phases (Fig. 3 – bottom phases, Fig. S1 – top phases). At the lowest amount of basic modulator of 45 µL, the dominant observed CNT type was (6,5), but two other chiralities of very low diameter emerged as well *i.e*. (6,4) 0.692 nm and (7,3) 0.705 nm. Their concentration in the parent mixture was very low judging on their absence in 2D PL plots and low absorbance of S_22_ and S_11_ features. Lack of sharpness of the signatures in Fig. 3a and a high level of noise corroborated that suspicion. As the volume of the introduced K_2_CO_3_ was increased to 75 µL, 90 µL and 135 µL light emission resulted exclusively from (6,5) CNTs. Related absorbance spectra (Fig. 3c) confirmed that predominantly the sample was composed of (6,5) CNTs along with some presence of (9,1) CNT, which commonly manifested along in this and other separation works since these species have the same diameter.

Once the K_2_CO_3_ volume was increased up to 225 µL, CNTs of (8,3) and (10,2) chiralities were found in the bottom phase. That indicated the shift of the diameter cut-off from 0.757 nm to 0.884 nm, which invalidated the desired partitioning.

**Figure 4:**
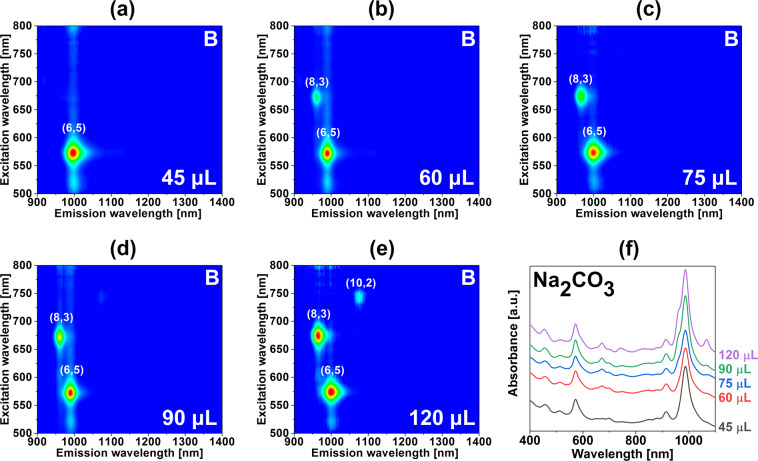
Characterization of the sorted material with the introduction of a chemical modulator (Na_2_CO_3_) into the ATPE system. 2D PL maps of the bottom phases upon addition of (**a**) 45 μL, (**b**) 60 μL, (**c**) 75 μL, (**d**) 90 μL and (**e**) 120 μL of Na_2_CO_3_ (10 wt%) per 4.59 mL total volume. (**f**) Corresponding absorbance spectra. Conditions of the experiments can be found in Table S3.

**Figure 5:**
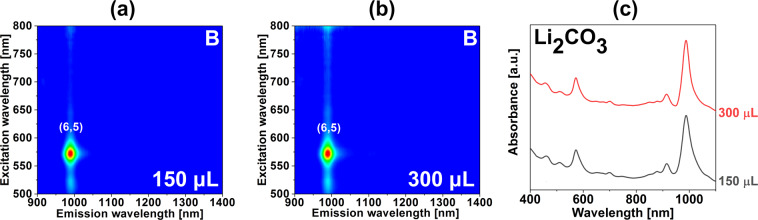
Characterization of the sorted material with the introduction of a chemical modulator (Li_2_CO_3_) into the ATPE system. 2D PL maps of the bottom phases upon addition of (**a**) 150 μL, and (**b**) 300 μL of Li_2_CO_3_ (1 wt%) per 4.59 mL total volume. (**f**) Corresponding absorbance spectra. Conditions of the experiments can be found in Table S4.

The following research showed that a similar effect could be obtained by the use of Na_2_CO_3_ (Fig. 4 – bottom phases, Fig. S2 – top phases). In this case, however, only the addition of 45 µL of the basic modulator enabled us to separate CNTs of (6,5) light emission characteristics (Fig. 5a). An increase in the volume of the added solution resulted in the gradual appearance of CNTs of other chiralities in the bottom phase. Namely, the introduction of 60 µL of Na_2_CO_3_ brought CNTs of (8,3) type to the bottom phase (Fig. 4b). When the volume was further increased to 120 µL also (10,2) CNTs could be detected (Fig. 4e). (8,3) and (10,2) CNTs are of 0.782 nm and 0.884 nm, respectively, which confirmed that the system worked through a “floating” diameter cut off mode. As always, despite the lack of (9,1) signatures in the excitation-emission PL maps, absorbance spectra of the samples unveiled its presence along with (6,5) CNTs. This once again confirmed that the partitioning in this approach was independent of the chiral angle of processed CNTs.

The obtained CNT types herein had a wide spectrum of chiral angles (6,5) – 27.00°, (8,3) – 15.30°, (10,2) – 8.95° yet they were isolated together due to similar diameters.

Such an outcome also resulted from the application of Li_2_CO_3_, but the volume of the employed modulator had to be significantly increased (Fig. 5 – bottom phases, Fig. S3 – top phases).

This was the consequence of low solubility of this chemical compound in water, because of which we could not prepare a 10 wt% solution, but we had to reduce the solute content down to 1 wt%. Nevertheless, both the addition of 150 µL and 300 µL of Li_2_CO_3_ 1 wt% aqueous solution led to the separation of (6,5) CNTs in the bottom phase (Fig. 5a,b) as in the previous experiments using K_2_CO_3_ and Na_2_CO_3_. The shape of the relevant absorbance spectra showed a high quality of the obtained material of predominantly (6,5) chiral angle (Fig. 5c).

**Figure 6:**
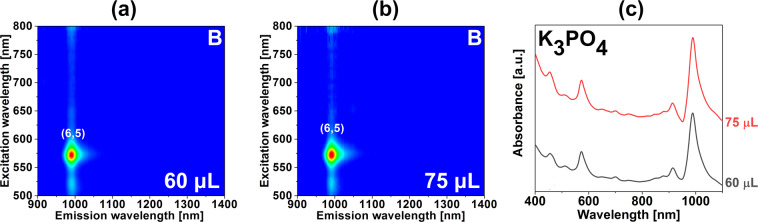
Characterization of the sorted material with the introduction of a chemical modulator (K_3_PO_4_) into the ATPE system. 2D PL maps of the bottom phases upon addition of (**a**) 60 μL, and (**b**) 75 μL of K_3_PO_4_ (10 wt%) per 4.59 mL total volume. (**f**) Corresponding absorbance spectra. Conditions of the experiments can be found in Table S5.

Finally, we decided to evaluate whether the ATPE system could work the same way with other anion causing the pH to rise to the desired level. For this purpose, we employed tribasic K_3_PO_4_ (Fig. 6 – bottom phases, Fig. S4 – top phases). Once more, appropriate adjustment of the necessary volume of the chemical modulator gave the targeted partitioning. Fractions of (6,5) CNT light emission characteristics were obtained by including 60 µL (Fig. 6) or 75 µL of the selected chemical compound (Fig. 6b). Sharp S_22_ and S_11_ transitions of the (6,5) CNTs visualized in the complementary absorbance spectra confirmed successful isolation (Fig. 6c).

**Figure 7:**
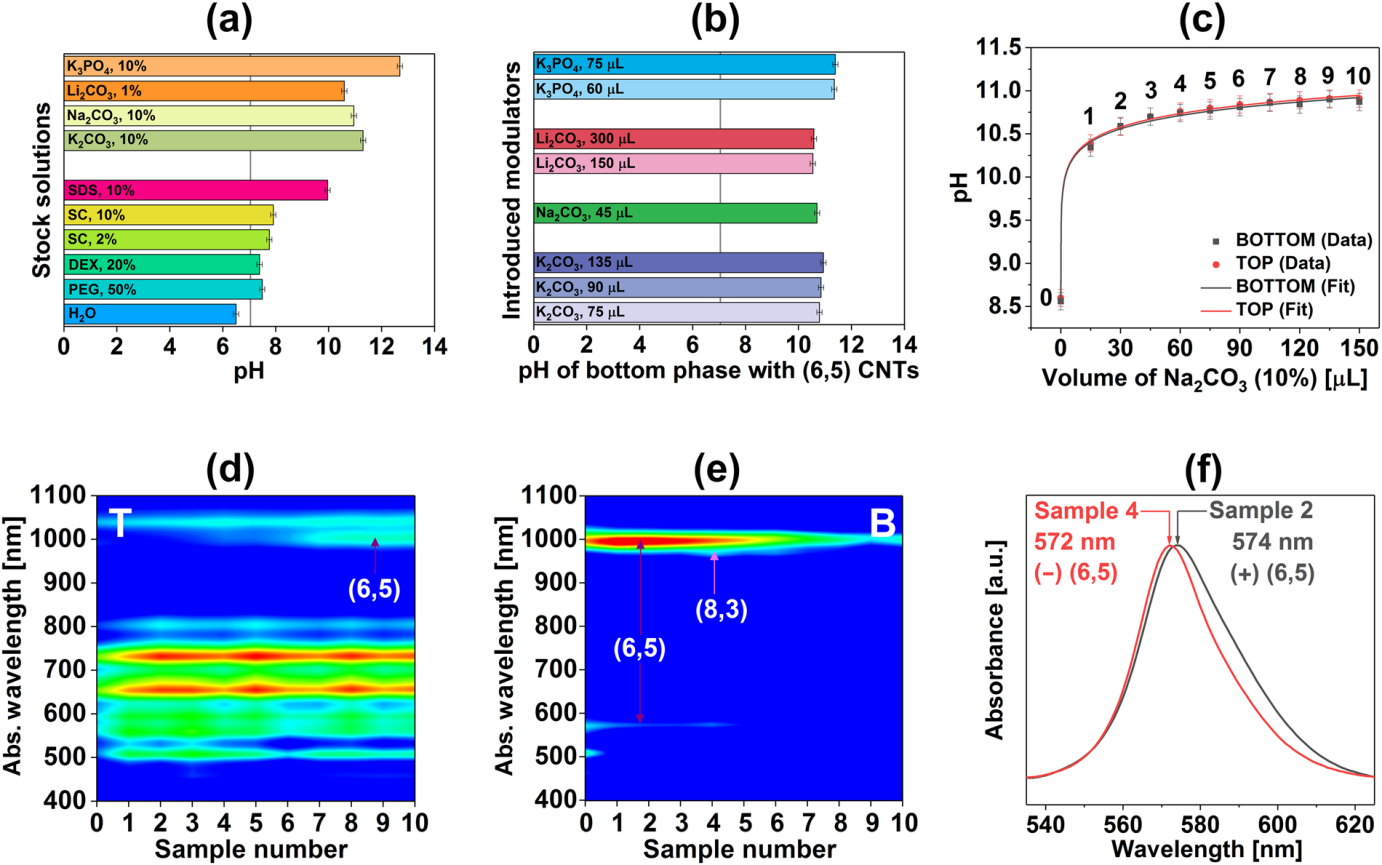
(**a**) pH of stock solutions used for ATPE separation, (**b**) pH of the bottom phase containing (6,5) CNTs upon introduction of the respective amount of chemical modulator to reach the partitioning, (**c**) pH of the top and bottom phases as a function of the introduced volume of Na_2_CO_3_ (10 wt%) per 4.59 mL total volume, normalized contour plots of the absorbance spectra of the (**d**) top, and (**e**) bottom phases as a function of the introduced volume of Na_2_CO_3_ (10 wt%), (**f**) magnification of the absorbance spectra of selected samples in the S_22_ area. Samples of the varying volume of Na_2_CO_3_ solution were numbered in panel (**c**) and then this notation was used in the subsequent plots (**d–f**). Conditions of the experiments can be found in Table S6.

All of the explored basic salts unequivocally enabled the separation of the low-diameter fraction of CNTs from the parent mixture (predominantly of (6,5) architecture). We decided to study how the pH of the ATPE separation system was affected by the selected modulators and other ingredients of the biphasic system (Fig. 7). First and foremost, it was interesting to uncover how different was the pH of the ATPE components from neutral (Fig. 7a). Solutions of PEG and DEX used to create the biphasic system had pH on the level of 7.49 and 7.39, respectively. SC showed a similar alkalinity of 7.76 or 7.91 for the 2 wt% or 10 wt% aq. formulations, respectively. Furthermore, the pH of the SDS solution was as high as 9.96. On the other hand, the pH of the employed water for the separation is slightly acidic (pH of 6.50) due to the dissolution of atmospheric CO_2_.

Chemical modulators used for this study confirmed to be even more basic. In the descending order, the pH values of their solutions were 12.69 (K_3_PO_4_, 10 wt%, aq.), 11.30 (K_2_CO_3_, 10 wt%, aq.), 10.95 (Na_2_CO_3_, 10 wt%, aq.) and 10.59 (Li_2_CO_3_, 1 wt%, aq.). Tribasic potassium phosphate exhibited much higher pH value because of a rather large difference between its pKa value and those of other basic salts. The least basic turned out to be the pH of lithium carbonate solution as a result of its tenfold lower concentration herein (and highest electronegativity, *vide infra*). This chemical compound was much less soluble in water by almost one and two orders of magnitude as compared with sodium and potassium carbonate, respectively, and hence we employed 1 wt%, aq. solution for this study as stressed before. On the other hand, the most basic of the carbonates was potassium carbonate at 10 wt% aq. solution. Its cation had the lowest electronegativity, and the largest size, which made it most susceptible to dissociation, and thus solvation^31,32^ which caused the observed effect.

Next, we analyzed the pH values of the bottom phases to elucidate which pH conditions led to the separation of CNT dispersions of exclusively (6,5) light emission characteristics (Fig. 7b). It was clear that the separation occurred at different pH values depending on the amount of introduced alkaline species. The amount and structure of salts were found to have an impact on the shape of micelles formed by various surfactants^33,34^ in other studies, which may justify the observed effect. It is important to note that introduced by us basic chemical modulators not only had a different electronic structure from one another, but they also came at various molar concentrations. As a consequence, they could affect the CNT hydration layer in a dissimilar fashion, which constituted the key factor for separation between the more hydrophobic PEG phase and more hydrophilic DEX medium.

To get a more detailed insight into the impact of a basic modulator on the course of ATPE separation we conducted “titration” of the ATPE system with selected for this purpose aqueous solution of sodium carbonate (Fig. 7c). In this set of experiments, each sample resulted from a separate separation routine carried out in parallel using a different volume of basic salt (to keep the volume of employed water of 4.59 mL in all separation routines). The results showed that the most gradual changes to the pH occurred at the very beginning as expected for a un-buffered solution. The pH of the top and bottom phases free of basic modulator were at ca. 8.60 (separation in the absence of modulator) and increased abruptly to ca. 10.40, 10.60, and 10.70 upon introduction of 15 µl, 30 µL, and 45 µL of Na_2_CO_3_, 10 wt%, aq, respectively. That corresponded to just 0.038 wt%, 0.075 wt% and 0.113 wt% of the basic salt content in the whole volume. Investigation of the optical properties (represented here as contour plots) of the resulting samples showed dynamic changes as a function of the modulator content (Fig. 7d,e). By monitoring the absorbance features of the CNT dispersions, we were able to track the progress of the separation as a function of pH. (6,5) CNTs were detected in the bottom phase from 0 µL up to 90 µL of Na_2_CO_3_ (pH range from 8.56 to 10.81, Samples 0 to 6). Synchronously, the relative amount of (6,5) CNTs in the top phase seemed to increase steadily from 45 µL of Na_2_CO_3_ upwards (pH range from 10.7 to 10.91, Samples 3 to 10) as species of gradually larger diameters were shifted into the bottom phase. The apparent increase of (6,5) content in the top phase in the presented data resulted from the fact that these two contour plots were normalized to the intensity of absorbance at 900 nm, at which point no CNT type would participate in the separation. It has to be noted that for separations in the presence of Na_2_CO_3,_ there was always some content of (6,5) CNTs in the top phase in contrast to for instance Li_2_CO_3_ and results of previous NH_3_ experiments^30^.

Lastly, a deeper analysis of the absorbance spectra of the isolated species suggested that depending on the pH different (6,5) enantiomers may have emerged in the bottom phase (Fig. 7f). Maxima of S_22_ signatures of (in all likelihood) predominantly (+) (6,5) and (−) (6,5) CNTs were detected at 574 nm (Sample 2, 30 µL of Na_2_CO_3_, pH of 10.59) and 572 nm (Sample 4, 60 µL of Na_2_CO_3_, pH of 10.74, which admittedly was contaminated with (8,3) CNTs), respectively. A similar shift was reported earlier in the literature indicative of different handedness of the resolved CNTs^35,36^. SC used for dispersion of CNTs in this study was reported to form a different coating structure on left- and right-handed enantiomers^35–37^, which affected the chemical nature of these species. That, in turn, influenced the partitioning course by the ATPE process and could result in the fractionation of optical isomers^28,38^. In our case, this justified the bimodal distribution of absorption signatures of CNTs of (6,5) chiralities peaking at conditions corresponding to Samples 1 and 2 (pH of 10.34 and 10.59), and then at those for Sample 4 (pH of 10.74). The boundary region corresponded to Sample 3 (pH of 10.70), which separated dispersions apparently rich in dextrorotatory and levorotatory (6,5) CNTs. To sum up, these separation conditions seemed to give dispersions of different enantiomeric excess of these two species. Further optimization of the separation conditions is required to reach chirality pure fractions for both samples to prove the hypothesis by circular dichroism.

## Conclusions

In summary, we demonstrated that simple inorganic compounds successfully modulated the course of CNT separation by the ATPE method. As a result, CNT fractions of (6,5) light emission characteristics were obtained in a single step. What is more, we indicated that left- and right-handed CNTs can be separated to some extent from one another by tailoring of the parameter space of this technique.

Our research showed that the pH of the biphasic system is important, but other factors may make a strong impact on the partitioning as well. The addition of extra chemical species into the process can tune the separation in a multidimensional way depending also on their structure and inherent electronic configuration. It should also be taken into consideration that the surfactant solutions commonly used for directing the separation have a strongly alkaline character. As a consequence, a gradual increase in their concentration in the multistep ATPE routine could be equivalent to titration under selected conditions.

We believe that further research is essential to obtain protocols for convenient isolation of a wider spectrum of CNT chiralities to pave the way towards the implementation of this material in real life. Ideally, such an approach should be fully reproducible, readily scalable and process higher relative weight of CNTs as compared with the total amount of the ATPE system, which is currently on the level of just 0.006 wt%. This, in turn, would greatly reduce the potential costs involved in the separation and eventually make the price of these promising chirality-controlled CNTs competitive on the market.

## Supplementary information

Supplementary information.
