# Supplementary material for: One-step sorting of single-walled carbon nanotubes using aqueous two-phase extraction in the presence of basic salts
Source: Sci Rep. 2020 Jun 8;10:9250. doi: 10.1038/s41598-020-66264-7 (PMC7280227; doi:10.1038/s41598-020-66264-7)
Supplement: Supplementary file 1 — Supplementary information. [file 41598_2020_66264_MOESM1_ESM.pdf]

Supplementary Information

One-step sorting of single-walled carbon nanotubes using aqueous two-phase extraction in the presence of basic salts

Blazej Podlesny[a], Tomohiro Shiraki[b], Dawid Janas[a,*]

[a] Department of Organic Chemistry, Bioorganic Chemistry and Biotechnology, Silesian University of Technology, B. Krzywoustego 4, 44-100 Gliwice, Poland

[b] Department of Applied Chemistry, Graduate School of Engineering, Kyushu University, 744 Motooka, Nishi-ku, 819-0395 Fukuoka, Japan

*Corresponding author: Dawid.Janas@polsl.pl

Table S1 ATPE parameters for separation of CNTs in the absence of chemical modulator

Compound	Aqueous concentration [wt%]	Volume [µL]	Composition of ATPE system [wt%]
DEX	20	1,350	6.757
PEG	50	540	6.757
SC	10	360	0.901
SDS	10	180	0.450
CNTs (in 2 wt% SC aq.)	0.1	225	0.006
H_2O	100	1,935	85.129
	TOTAL	4,590	100

Table S2 ATPE parameters for separation of CNTs in the presence of K_2CO_3

**Compound**	**Aqueous concentration [wt%]**	**Volume [µL]**				
DEX	20	1,350	1,350	1,350	1,350	1,350
PEG	50	540	540	540	540	540
SC	10	360	360	360	360	360
SDS	10	180	180	180	180	180
K_2CO_3	10	45	75	90	135	225
CNTs (in 2 wt% SC aq.)	0.1	225	225	225	225	225
H_2O	100	1,890	1,860	1,845	1,800	1,710
	TOTAL	4,590	4,590	4,590	4,590	4,590

which corresponds to

**Compound**	**Aqueous concentration [wt%]**	**Composition of ATPE system [wt%]**				
DEX	20	6.765	6.770	6.772	6.780	6.795
PEG	50	6.765	6.770	6.772	6.780	6.795
SC	10	0.902	0.902	0.903	0.904	0.906
SDS	10	0.451	0.451	0.451	0.452	0.453
K_2CO_3	10	0.113	0.188	0.226	0.339	0.566
CNTs (in 2 wt% SC aq.)	0.1	0.006	0.006	0.006	0.006	0.006
H_2O	100	84.999	84.913	84.869	84.739	84.478

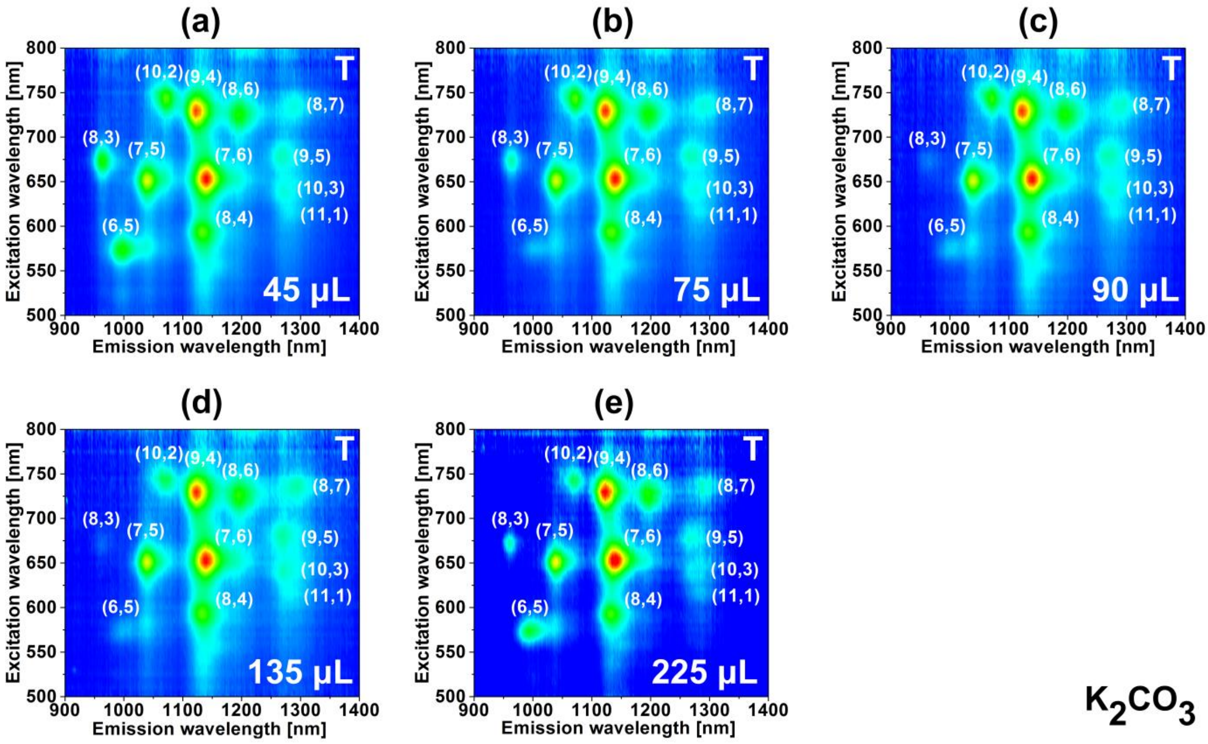

Figure S1 Characterization of the sorted material with the introduction of a chemical modulator (K_2CO_3) into the ATPE system. 2D PL maps of the top phases upon addition of (a) 45 µl, (b) 75 µL, (c) 90 µL, (d) 135 µL and (e) 225 µL of K_2CO_3 (10 wt%) per 4.59 mL total volume.

Table S3 ATPE parameters for separation of CNTs in the presence of Na_2CO_3

Compound	Aqueous concentration [wt%]	Volume [µL]				
DEX	20	1,350	1,350	1,350	1,350	1,350
PEG	50	540	540	540	540	540
SC	10	360	360	360	360	360
SDS	10	180	180	180	180	180
Na_2CO_3	10	45	60	70	90	120
CNTs (in 2 wt% SC aq.)	0.1	225	225	225	225	225
H_2O	100	1,890	1,875	1,865	1,845	1,815
	TOTAL	4,590	4,590	4,590	4,590	4,590

which corresponds to

Compound	Aqueous concentration [wt%]	Composition of ATPE system [wt%]				
DEX	20	6.765	6.767	6.769	6.772	6.777
PEG	50	6.765	6.767	6.769	6.772	6.777
SC	10	0.902	0.902	0.903	0.903	0.904
SDS	10	0.451	0.451	0.451	0.451	0.452
Na_2CO_3	10	0.113	0.150	0.175	0.226	0.301
CNTs (in 2 wt% SC aq.)	0.1	0.006	0.006	0.006	0.006	0.006
H_2O	100	84.999	84.956	84.927	84.869	84.783

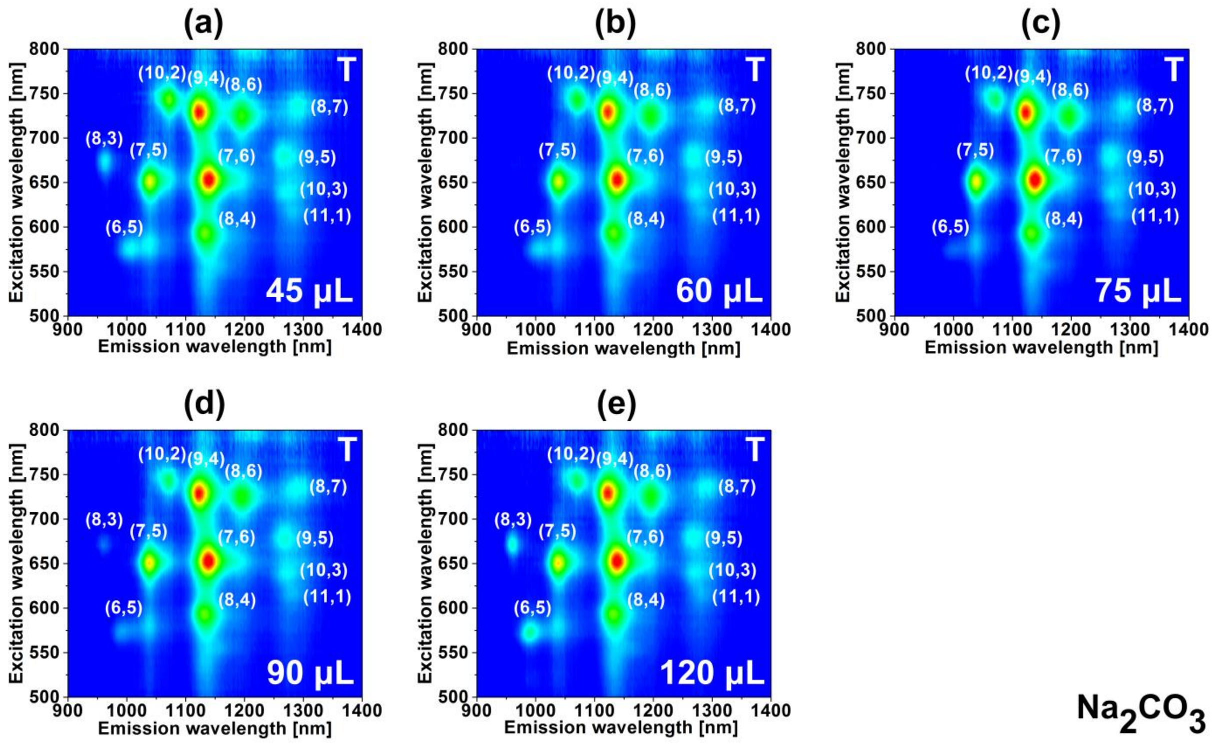

Figure S2 Characterization of the sorted material with the introduction of a chemical modulator (Na_2CO_3) into the ATPE system. 2D PL maps of the bottom phases upon addition of (a) 45 µl, (b) 60 µL, (c) 75 µL, (d) 90 µL and (e) 120 µL of Na_2CO_3 (10 wt%) per 4.59 mL total volume. (f) Corresponding absorbance spectra.

Table S4 ATPE parameters for separation of CNTs in the presence of Li_2CO_3

Compound	Aqueous concentration [wt%]	Volume [µL]	
DEX	20	1,350	1,350
PEG	50	540	540
SC	10	360	360
SDS	10	180	180
Li_2CO_3	1	150	300
CNTs (in 2 wt% SC aq.)	0.1	225	225
H_2O	100	1,785	1,635
	TOTAL	4,590	4,590

which corresponds to

Compound	Aqueous concentration [wt%]	Composition of ATPE system [wt%]	
DEX	20	6.760	6.762
PEG	50	6.760	6.762
SC	10	0.901	0.902
SDS	10	0.451	0.451
Li_2CO_3	1	0.038	0.075
CNTs (in 2 wt% SC aq.)	0.1	0.006	0.006
H_2O	100	85.086	85.042

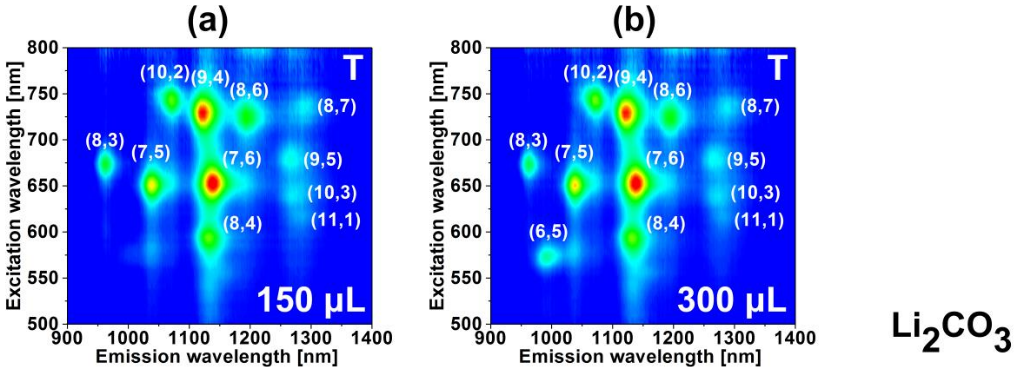

Figure S3 Characterization of the sorted material with the introduction of a chemical modulator (Li_2CO_3) into the ATPE system. 2D PL maps of the bottom phases upon addition of (a) 150 µl, and (b) 300 µL of Li_2CO_3 (1 wt%) per 4.59 mL total volume. (f) Corresponding absorbance spectra.

Table S5 ATPE parameters for separation of CNTs in the presence of K_3PO_4

Compound	Aqueous concentration [wt%]	Volume [µL]	
DEX	20	1,350	1,350
PEG	50	540	540
SC	10	360	360
SDS	10	180	180
K_3PO_4	10	60	75
CNTs (in 2 wt% SC aq.)	0.1	225	225
H_2O	100	1,875	1,860
	TOTAL	4,590	4,590

which corresponds to

Compound	Aqueous concentration [wt%]	Composition of ATPE system [wt%]	
DEX	20	6.767	6.770
PEG	50	6.767	6.770
SC	10	0.902	0.903
SDS	10	0.451	0.451
K_3PO_4	1	0.150	0.188
CNTs (in 2 wt% SC aq.)	0.1	0.006	0.006
H_2O	100	84.956	84.913

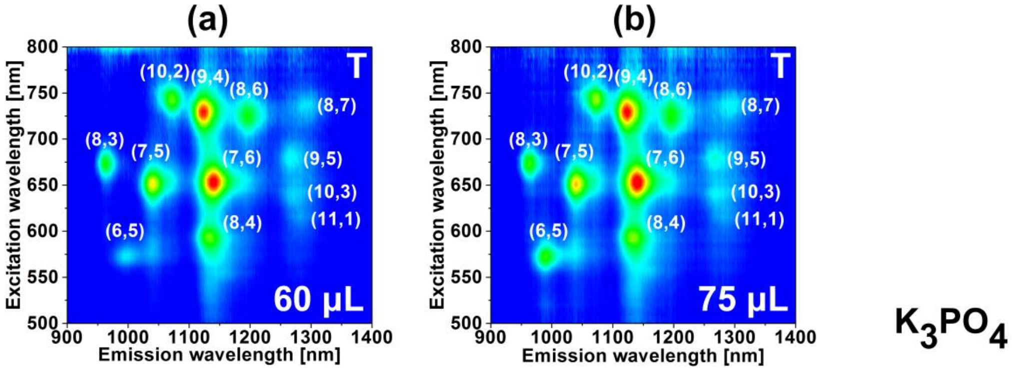

Figure S4 Characterization of the sorted material with the introduction of a chemical modulator (K_3PO_4) into the ATPE system. 2D PL maps of the bottom phases upon addition of (a) 60 µl, and (b) 75 µL of K_3PO_4 (10 wt%) per 4.59 mL total volume. (f) Corresponding absorbance spectra.

Table S6 ATPE parameters used for titration with Na_2CO_3

Compound	Aqueous concentration [wt%]	Volume [µL]				
DEX	20	1,350	1,350	1,350	1,350	1,350
PEG	50	540	540	540	540	540
SC	10	360	360	360	360	360
SDS	10	180	180	180	180	180
Na_2CO_3	10	0	15	30	45	60
CNTs (in 2 wt% SC aq.)	0.1	225	225	225	225	225
H_2O	100	1,935	1,920	1,905	1,890	1,875
	TOTAL	4,590	4,590	4,590	4,590	4,590
	Sample no.	(0)	(1)	(2)	(3)	(4)

which corresponds to

Compound	Aqueous concentration [wt%]	Composition of ATPE system [wt%]				
DEX	20	6.757	6.760	6.762	6.765	6.767
PEG	50	6.757	6.760	6.762	6.765	6.767
SC	10	0.901	0.901	0.902	0.902	0.902
SDS	10	0.450	0.451	0.451	0.451	0.451
Na_2CO_3	10	0.000	0.038	0.075	0.113	0.150
CNTs (in 2 wt% SC aq.)	0.1	0.006	0.006	0.006	0.006	0.006
H_2O	100	85.129	85.086	85.042	84.999	84.956

and

Compound	Aqueous concentration [wt%]	Volume [µL]				
DEX	20	1,350	1,350	1,350	1,350	1,350
PEG	50	540	540	540	540	540
SC	10	360	360	360	360	360
SDS	10	180	180	180	180	180
Na_2CO_3	10	75	90	105	120	135
CNTs (in 2 wt% SC aq.)	0.1	225	225	225	225	225
H_2O	100	1,860	1,845	1,830	1,815	1,800
	TOTAL	4,590	4,590	4,590	4,590	4,590
	Sample no.	(5)	(6)	(7)	(8)	(9)

which corresponds to

Compound	Aqueous concentration [wt%]	Composition of ATPE system [wt%]				
DEX	20	6.770	6.772	6.775	6.777	6.780
PEG	50	6.770	6.772	6.775	6.777	6.780
SC	10	0.903	0.903	0.903	0.904	0.904
SDS	10	0.451	0.451	0.452	0.452	0.452
Na_2CO_3	10	0.188	0.226	0.263	0.301	0.339
CNTs (in 2 wt% SC aq.)	0.1	0.006	0.006	0.006	0.006	0.006
H_2O	100	84.913	84.869	84.826	84.783	84.739

and

Compound	Aqueous concentration [wt%]	Volume [µL]
DEX	20	1,350
PEG	50	540
SC	10	360
SDS	10	180
Na_2CO_3	10	150
CNTs (in 2 wt% SC aq.)	0.1	225
H_2O	100	1,785
	TOTAL	4,590
	Sample no.	(10)

which corresponds to

Compound	Aqueous concentration [wt%]	Composition of ATPE system [wt%]
DEX	20	6.783
PEG	50	6.783
SC	10	0.904
SDS	10	0.452
Na_2CO_3	10	0.377
CNTs (in 2 wt% SC aq.)	0.1	0.006
H_2O	100	84.696
